# Corrigendum

**DOI:** 10.7448/IAS.17.1.19328

**Published:** 2014-08-08

Regarding the paper ‘Achieving universal access and moving towards elimination of new HIV infections in Cambodia’ by Mean Chhi Vun, Masami Fujita, Tung Rathavy, Mao Tang Eang, Seng Sopheap, Samreth Sovannarith, Chhea Chhorvann, Ly Vanthy, Oum Sopheap, Emily Welle, Laurent Ferradini, Chin Sedtha, Sok Bunna and Robert Verbruggen

Published in *Journal of the International AIDS Society.* Citation: http://www.jiasociety.org/index.php/jias/article/view/18905 | http://dx.doi.org/10.7448/IAS.17.1.18905

Figure 5 source information was not included.

The corrected paper can be found below:
