# Supplementary material for: Corrigendum
Source: J Int AIDS Soc. 2014 Aug 8;17(1):19328. doi: 10.7448/IAS.17.1.19328 (PMC4127810; doi:10.7448/IAS.17.1.19328)
Supplement: Related texts

# Sub article: Achieving universal access and moving towards elimination of new HIV infections in Cambodia

**DOI:** 10.7448/IAS.17.1.18905

**Published:** 2014-06-19

**Authors:** Mean Chhi Vun, Masami Fujita, Tung Rathavy, Mao Tang Eang, Seng Sopheap, Samreth Sovannarith, Chhea Chhorvann, Ly Vanthy, Oum Sopheap, Emily Welle, Laurent Ferradini, Chin Sedtha, Sok Bunna, Robert Verbruggen

**Affiliations:** 1 National Centre for HIV/AIDS Dermatology and STDs, Ministry of Health, Phnom Penh, Cambodia; 2 World Health Organization, Phnom Penh, Cambodia; 3 National Maternal and Child Health Centre, Ministry of Health, Phnom Penh, Cambodia; 4 National Centre for Tuberculosis and Leprosy Control, Ministry of Health, Phnom Penh, Cambodia; 5 United States Centres for Disease Control and Prevention, Global AIDS Program, Phnom Penh, Cambodia; 6 KHANA, Phnom Penh, Cambodia; 7 Clinton Health Access Initiative, Phnom Penh, Cambodia; 8 FHI 360, Phnom PenhCambodia; 9 UNICEF, Phnom Penh, Cambodia; 10 United States Agency for International Development, Phnom Penh, Cambodia; 11 UNAIDS, Phnom Penh, Cambodia

**Keywords:** HIV, response, epidemic, universal access, service linkage, integration

## Abstract

**Introduction:**

In the mid-1990s, Cambodia faced one of the fastest growing HIV epidemics in Asia. For its achievement in reversing this trend, and achieving universal access to HIV treatment, the country received a United Nations millennium development goal award in 2010. This article reviews Cambodia’s response to HIV over the past two decades and discusses its current efforts towards elimination of new HIV infections.

**Methods:**

A literature review of published and unpublished documents, including programme data and presentations, was conducted.

**Results and discussion:**

Cambodia classifies its response to one of the most serious HIV epidemics in Asia into three phases. In Phase I (1991–2000), when adult HIV prevalence peaked at 1.7% and incidence exceeded 20,000 cases, a nationwide HIV prevention programme targeted brothel-based sex work. Voluntary confidential counselling and testing and home-based care were introduced, and peer support groups of people living with HIV emerged. Phase II (2001–2011) observed a steady decline in adult prevalence to 0.8% and incidence to 1600 cases by 2011, and was characterized by: expanding antiretroviral treatment (coverage reaching more than 80%) and continuum of care; linking with tuberculosis and maternal and child health services; accelerated prevention among key populations, including entertainment establishment-based sex workers, men having sex with men, transgender persons, and people who inject drugs; engagement of health workers to deliver quality services; and strengthening health service delivery systems. The third phase (2012–2020) aims to attain zero new infections by 2020 through: sharpening responses to key populations at higher risk; maximizing access to community and facility-based testing and retention in prevention and care; and accelerating the transition from vertical approaches to linked/integrated approaches.

**Conclusions:**

Cambodia has tailored its prevention strategy to its own epidemic, established systematic linkages across different services and communities, and achieved nearly universal coverage of HIV services nationwide. Still, the programme must continually (re)prioritize the most effective and efficient interventions, strengthen synergies between programmes, contribute to health system strengthening, and increase domestic funding so that the gains of the previous two decades are sustained, and the goal of zero new infections is reached.

## Introduction

Situated in the heart of South East Asia, Cambodia is a low-income country that is recovering from several decades of regional and internal conflict. Its population of 14.86 million [1] is growing at a rate of 1.54% with 80% living in rural areas and 20% dwelling in cities [2]. With a life expectancy of 57 years for men and 65 years for women, gross domestic product is [estimated at] 946 United States dollars per capita purchasing power parity [3]. From 2000 to 2010, total fertility reduced from 4.0 to 3.0 children per woman, and child mortality fell from 95 to 45 infant deaths per 1000 live births and 124 to 54 deaths of under-fives per 1000 live births [4]. Maternal mortality dropped from 472 to 206 deaths to 100,000 live births between 2005 and 2010 [4]. While Cambodia’s health indicators have been improving, noteworthy progress has also been made in reducing HIV prevalence and incidence, and achieving universal access to antiretroviral treatment (ART).

The first HIV infection was officially detected in 1991 during screening of blood donors [5], at which time HIV was spreading rapidly throughout Asia. Initially concentrated within the sex industry, the epidemic expanded from female sex workers^1^ to their male clients, to the spouses of these men and to their newborns. Incident infections exceeded 20,000 cases in 1995 and prevalence peaked at 1.7% among adults (15–49 years) in 1998–1999 [6] but since the introduction of an anti-trafficking law that caused a shift of sex workers away from brothel-based work and into the entertainment industry, the modes of transmission have gradually shifted. Transmission via sex work has since declined, and other key populations are now experiencing HIV infections. These populations include people who inject drugs [7], men who have sex with men and transgender (TG) persons. Today, almost half of HIV transmissions are estimated to occur among couples and through casual sex [6].

Despite the 53% growth of Cambodia’s population from 9.72 in 1992 to 14.86 million in 2012 [1], annual new infections have dropped 20-fold (Figure 1). Annual AIDS-related deaths have reduced by two thirds over the past 10 years, and adult prevalence declined to 0.7% by 2012 [6]. In 2010, Cambodia received a millennium development goal (MDG) award from the United Nations as a global recognition of the country’s national leadership, commitment to and progress in working towards halting and reversing the spread of HIV, and achieving universal access to HIV treatment [8].

**Figure 1:** Trend of the epidemic. Estimated and projected numbers of PLHIV, new HIV infections and modes of HIV transmission in Cambodia [6].

This article reviews Cambodia’s response to HIV over the past two decades, concentrating on the strategic decisions taken and the many efforts that dealt vigorously with the epidemic. Describing all three phases of the HIV response, the article outlines Cambodia’s current efforts to achieve the elimination of new infections.

## Methods

This article is based on a literature review of available data, documents and reports (published and unpublished), corroborated by the personal insights and experiences of the authors.

## Results and discussion

Health services in Cambodia are delivered via two facility types: health centres providing basic services such as antenatal care (ANC), delivery, diagnosis and treatment of tuberculosis (TB) and referral hospitals (at national, provincial and district level) providing a broader range of clinical care including ART and laboratory services. Operational districts, which are clusters of administrative districts, oversee health service management. There are 80 operational districts across Cambodia’s 25 provinces, and each contains at least one referral hospital.

The National Centre for HIV/AIDS, Dermatology and STI (NCHADS) is the body within the Ministry of Health responsible for the health sector response to HIV and other sexually transmitted diseases in Cambodia. As such, it collaborates with relevant departments and centres of the Ministry of Health, the National AIDS Authority and other government institutions, as well as health service providers, non-governmental and other civil society organizations, and development partners.

### Evolution of the health sector response to HIV

Cambodia classifies its response to one of the most serious HIV epidemics in Asia into three phases (Table 1).

**Table 1:** The three phases of the health sector response to HIV: key features and milestones

	Phase I (1991–2000)	Phase II (2001–2011)	Phase III (2012–2020)
HIV epidemic	Adult prevalence peaked at 1.7% in 1998 Most new infections from brothel sex work	Adult prevalence declined to 0.8% in 2011 Sex work shifted to entertainment establishments MSM/TG/PWID emerged	1350 new infections in 2012 - 50% partners and casual - 40% key populations - 10% MTCT
HIV prevention for key populations	100% condom use for brothel-based sex workers IEC for general population	Peer outreach to entertainment workers and other emerging key populations, with referral to health services	Sharpening response to key populations at higher risk
Testing and counselling	VCCT in main cities	Nationwide expansion of VCCT in all operational districts and 20% of health centres PITC in most health centres	Finger-prick HIV testing and counselling in all health centres and outreach settings Testing of partners of PLHIV and key populations
Care and treatment including PMTCT	Extensive home-based care PLHIV peer support groups and network emerged	ART scale-up Expanding PMTCT and TB/HIV	Maximizing retention throughout cascades including partner testing and TB/HIV Test and treat Elimination of MTCT
Strategic information	Sentinel surveillance	Electronic database for each service Continuous quality improvement	Client tracking across services Monitoring impact HIV drug resistance
Orientation of programme management	Vertical	Linkage	Integration

IEC=information, education, communication; MTCT=mother-to-child transmission; MSM=men who have sex with men; PITC=provider-initiated testing and counselling; PMTCT=prevention of mother-to-child transmission; PWID=people who inject drugs; VCCT=voluntary confidential counselling and testing; TG=transgender.

#### Phase I. Intensive response to the source of the epidemic (1991–2000)

Phase I took place while the country was recovering from the previous decades of national instability, when HIV was expanding most rapidly. Unprotected sex work was fuelling the spread of HIV and other STI [9]. The rate of commercial sex use among moto-taxi drivers peaked at 62% in 1998 [6] while their reported consistent condom use in the past three months was also 62%. HIV prevalence among brothel-based sex workers remained higher than 40% throughout the decade [6]. Thus, early interventions were focused on preventing HIV and STI transmission through brothel-based sex work.

In the early to mid-1990s, social marketing of condoms, peer education and outreach were directed at sex workers. Then in 1999, the Prime Minister of Cambodia adopted the 100% Condom Use Programme (CUP), and it was rolled out nationwide [10]. It was implemented with an emphasis on preventing HIV and STIs among brothel-based sex workers and their clients. All sex establishments had to participate in 100% CUP so that clients had nowhere to purchase sex without using condoms. Notably, the programme had the close collaboration of brothel owners, local authorities and health workers, particularly those involved in STI services, which was key to its success [11].

Three key structures were established in the mid-1990s to create an environment in which people living with HIV (PLHIV) could seek health and social support services. First, in 1995, Voluntary Confidential Counselling and Testing (VCCT) sites were introduced. While not yet “linked” to other service delivery points, their co-location with health facilities became conducive to access to treatment. Second, home-based care (HBC) networks were formed, comprising health centre staff and volunteers, many of whom were PLHIV. Third, PLHIV peer support groups and networks emerged from the HBC network, helping members cope with the social and emotional aspects of living with HIV.

During the latter half of Phase I, the trends among direct brothel-based sex workers in Phnom Penh began improving; self-reported condom use at last paid intercourse increased from less than 40% to consistently over 90%, STI prevalence halved and HIV prevalence started to decline from 42% [6] (Figure 2). By 2001, moto-taxi drivers’ use of commercial sex workers had dropped to 29%, and reported consistent condom use with sex workers in the past three months had increased to 84% [6]. These early efforts to respond to the epidemic, including the establishment of supportive health and social structures, laid a good foundation for Phase II.

**Figure 2:** HIV, condom use and STI. Trends among brothel-based sex workers, Phnom Penh.

#### Phase II. Rapid expansion of HIV health services (2001–2011)

In the second Phase of Cambodia’s response, there was rapid scale-up of HIV counselling, testing, care and treatment (Table 2). Systematic linkages were created with the national maternal and child health (MCH) and TB programmes, and linkages between health facilities and the community were strengthened. Growing attention was given to key populations, to the quality of service delivery and to patient outcomes. The six strategic components of this phase are described below.

**Table 2:** Expansion of services and coverage throughout Phase II

	2001	2003	2005	2007	2009	2011
Estimated no. of PLHIV	107,371	103,423	96,550	90,176	85,885	83,413
No. of health centres (HC)*	(850)	n.a.	n.a.	957	967	1004
VCCT sites	25	51	109	197	233	255
ART – no. of operational districts with ART sites	1	4	32	49	52	55
No. of PLHIV receiving ART	71	2230	12,355	26,664	37,315	46,481
Estimated no. of PLHIV needing ART†	31,537	38,644	42,039	44,348	47,576	58,216
% PLHIV receiving ART/estimated no. of PLHIV needing ART	0.2%	5.8%	29%	60%	78%	80%
Home-based care (HBC) – no. of HBC teams	52	n.a.	261	253	328	354
No. of HC with HBC team	n.a.	n.a.	366	683	742	841
No. of operational districts with HBC teams	n.a.	n.a.	n.a.	n.a.	67	72
PMTCT – no. of HC offering HIV testing to pregnant women	0	7	25	91	228	944
No. of pregnant women tested for HIV	n.a.	18,553	46,468	95,277	144,236	314,953
% Pregnant women tested for HIV/estimated pregnant women	n.a.	5.8%	14.2%	28.2%	44.0%	86.0%
No. of HIV+ pregnant women receiving ARV^ for PMTCT	n.a.	87	175	599	798	1014
% HIV+ pregnant women receiving ARV^ for PMTCT/HIV+ pregnant women	n.a.	3.5%	9.0%	11.2%	32.3%	69.2%
TB/HIV – no. of operational districts offering HIV testing to TB cases	n.a.	9	15	52	74	77
% PLHIV screened for TB upon pre-ART enrolment	n.a.	n.a.	n.a.	n.a.	34.5%	85.0%
No. of TB cases tested for HIV	n.a.	n.a.	n.a.	17,105	28,246	32,544
% TB cases tested for HIV/TB cases	n.a.	n.a.	n.a.	4.7%	70.3%	82.0%
No. of HIV+ TB cases receiving ART	n.a.	n.a.	n.a.	610	526	1083
% HIV+ TB cases receiving ART/HIV-positive TB cases	n.a.	n.a.	n.a.	11.0%	14.6%	79.2%

* Reporting of the number of health centres was established in 2007. The figure for 2001 is an estimate;

† ART eligibility criteria for adults changed in 2010 from CD4 count 200 to 350; ^ARV for PMTCT includes single dose nevirapine.

^ ARV for PMTCT includes single dose nevirapine.

#### Decentralizing ART services to district level, and VCCT to district and health centre levels

When expansion of ART services emerged as a national agenda in 2002, the country had only a few isolated ART services at national level hospitals with specialized clinicians. Local hospitals were not fully functional, as the nation was still recovering from decades of conflict. NCHADS led extensive consultations among key stakeholders to determine how to manage the expansion, which resulted in the decision to decentralize treatment services to operational district level. Providing services at this level would build on and support the extensive network of HBC and PLHIV peer support groups (described in the next section).

VCCT was expanded from less than 20 sites in Phnom Penh and some provincial towns to all operational districts, and later into one in five health centres. As the expansion data in Table 1 show, rolling out this service made possible the various linkages that would follow, including the continuum of care, a linking model for prevention of mother-to-child transmission (PMTCT) and TB/HIV, and outreach to key populations and referral to health services.

A rapid test-based algorithm was implemented throughout VCCT services, and provider-initiated testing and counselling (PITC) was made available to pregnant women and TB cases in health centres. Procedures for referring blood samples to VCCT sites were established, since health centres without VCCT were not equipped with test kits. Key populations were targeted and referred in person by non governmental organizations (NGOs) outreach teams to VCCT twice per year. Outreach testing by VCCT staff was also explored late during this phase.

#### Establishing the continuum of care

While the strategy for ART expansion was being conceptualized, NCHADS and other stakeholders noted the many barriers to PLHIV accessing services. Formal and informal dialogue with PLHIV peer support group members highlighted the burdens of stigma, discrimination, and financial, social and psychological pressures. Having explored a range of service delivery models through extensive consultation, a single model was adopted whereby each operational district was to forge partnerships between public health sector facilities, PLHIV networks, NGOs and the community. To guide this process and lay the foundation for systematic expansion of a standard service package, the Continuum of Care framework [12] was developed in 2003. Figure 3 shows the 2011 framework, an updated version that reflected the evolution of services.

**Figure 3:** Continuum of Care for PLHIV (2011 Framework). Continuum of Care refers to a network of resources and services across homes, the community and health facilities that provide long-term comprehensive support for those in need.

The Continuum of Care Framework was applied at the operational district level, where the Vice Governor and the Referral Hospital Director or pre-ART/ART Team Leader co-chaired the Continuum of Care Coordination Committee. This leadership ensured coordination of stakeholders and facilitated communication among them. Meetings were well attended by representatives from health facilities, NGOs, PLHIV and the local community, which was also a key to their success.

After being involved in HBC teams for a time, health centre staff were instructed to give up their membership because of the time commitments that impinged on their facility-based duties. Self-help groups subsequently grew to play a major role in supporting HBC, and the two networks were responsible for encouraging those who were at high risk of HIV infection (such as spouses and children of PLHIV) to access HIV testing and counselling (HTC). HIV-positive cases were linked to care and treatment services at operational district-level referral hospitals, where PLHIV volunteers would help them enrol in pre-ART care. Conversely, hospitals referred patients back to HBC teams and self-help groups to ensure that they were supported in their communities.

Attached to pre-ART/ART services, Centres for Friends Helping Friends (Mondul Mith Chuoy Mith, or MMM) also played a key role in facilitating social support and community education. MMM services were equally extended to children where paediatric AIDS care services were available on the paediatric ward (designated as mmm). With facilities located within the compound of the hospital, MMM/mmm was understood to be the space in which connections could be made among PLHIV, families, NGOs, health workers, religious leaders and local authorities. As such, MMM/mmm activities contributed significantly to the improvement of the health and wellbeing of PLHIV, as well as to the reduction of stigma and discrimination.

The Continuum of Care coordination meetings and MMM/mmm successfully aligned with health facilities and community structures, and so the standard model laid out in the framework was expanded progressively across the nation, together with ART. The expansion process was guided by consideration of factors such as geographic distribution of disease burden, projected capacity of referral hospitals, and existence of HBC and PLHIV support groups.

#### Establishing service linkages in collaboration with MCH and TB programmes

After the late 1990s, several unsystematic attempts were made to introduce prevention of mother-to-child transmission and TB/HIV collaborative activities [13]. At the time, VCCT was offered at hospitals while maternal and child health and TB services were available at the health centre level. A several-year long trial period of coordinating HIV, MCH and TB services did not lead to substantive service coverage.

By 2006, it was clear that collaboration among HIV, MCH and TB programmes would succeed if an agreed service delivery model explicitly prescribed linkages between the programmes. The roles and responsibilities of respective programmes were identified through concerted efforts to establish systematic linkages between the concerned services. In particular, it was important to situate PITC at health centre level, as this was where pregnant women presented for ANC and where TB cases were being seen. As such, there was a major breakthrough in collaborative service delivery.

For PMTCT, the “Linked Response” [14] strategy was developed. It consisted of: ANC initiating HTC among pregnant women at health centres; systematic referral services to confirmatory HIV testing at VCCT sites; Antiretroviral medicine (ARV) prophylaxis or treatment at ART sites at referral hospitals; maternity care; exposed infant care; and paediatric HIV services. This model built on the continuum of care strategy which focused on access to ART and its retention. While use of rapid tests for HIV testing was expanded to referral hospitals and several health centres with VCCT, most health centres where ANC services were offered were not equipped with such capacity, nor with HIV test kits. Therefore, the system of blood sample referral from health centres to a nearby health centre with VCCT or a referral hospital was developed. It was first implemented in one operational district (Figure 4), then promptly demonstrated in five operational districts. These referrals were a key feature of the Linked Response that resulted in a rapid and sustained uptake of HIV testing through ANC. To help other testing services reach “universal” coverage, syphilis testing was added in the following year (Figure 5).

**Figure 4:** Linked Response demonstration sites in Kirivong operational district, 2008. Left, before Linked Response was introduced, pregnant women seen at 20 health centres (HC) had to travel to only two VCCT sites and one OI/ART service. Right, Linked Response introduced five new VCCT sites at the health centre level and a blood sample referral system to reduce the number of in-person referrals, travel distances and delays.

**Figure 5:** Impact of Linked Response. Percentage of pregnant women attending ANC tested for HIV/syphilis, out of total number of expected pregnant women. Testing coverage spiked in five operational districts [15].

This experience gave substantive justification to scaling up the “Linked Response” nationwide, and resources were readily mobilized towards that end. Subsequently, coverage of PMTCT cascades, HIV testing and triple ARV prophylaxis or ART in pregnant women improved dramatically.

Throughout the decade, the national HIV and TB programmes worked on developing a framework for collaboration [15]; TB/HIV collaborative activities [16] and cross-referral systems were piloted, and standard operating procedures were outlined for HIV testing of TB patients and vice versa [17]. In many referral hospitals, the HIV treatment team had a TB physician screen PLHIV. The reciprocal referral of TB patients’ blood samples for HIV testing was introduced at health centres where there was no VCCT service. Referring samples was found to be more efficient than referring patients or using mobile testing units. Thus, its expansion started in 2009.

Together, the respective linking of HIV with MCH and TB services resulted in progressive coverage of HIV testing of pregnant women and TB cases to over 80% in 2011. Also by 2011, coverage of ARV (including single dose nevirapine) among estimated HIV-positive pregnant women had reached 69.2%. Of all newly diagnosed TB cases with HIV infections in 2012, 89% received ART. However, pregnant women and TB cases were not able to receive the same day test results under this model. In the cumbersome referral process of linking first test, confirmatory test, pre-ART care enrolment and other elements of PMTCT and TB/HIV services, substantive drop-outs and delays in accessing treatment were observed.

#### Responding to changing epidemics

In 2008, the Trafficking Law was revised to prohibit the public solicitation, procurement and management of an establishment for prostitution and the provision of premises for prostitution [18]. While it did not prohibit engaging in prostitution, local interpretation of the law led to the closure of brothels as well as crackdowns on sex workers and their dispersal into entertainment establishments such as beer gardens, karaoke clubs, massage parlours and “underground” [19–21]. Consequently, the official terminology was changed from “sex workers” to “entertainment workers.” It was reported that HIV prevention efforts among the key populations and their access to health care were disrupted [22–24].

Then the global financial crisis, which affected Cambodia in 2009, resulted in loss of employment for around 50,000 garment workers. Roughly 14,000 female workers entered the entertainment sector at this time [23, 25].

When the Commune/Village Safety Policy was introduced in 2011, utilization of HIV services by high-risk groups [26] is reported to have been further affected, as the policy urges commune authorities to ensure that communes are free of prostitution, theft, drugs, violence and various other threats to safety.

Meanwhile, rising HIV transmission was noticed among men who have sex with men, TG persons, and people who use and/or inject drugs. Reaching all of these populations required new, more innovative approaches.

To respond to the changing situation, NCHADS, implementing NGOs and partners developed a new approach in 2009, called the Continuum of Prevention to Care and Treatment for key populations [27]. This new approach engaged NGOs and peer networks to reach key populations at entertainment establishments and hot spots, to provide them with education and prevention commodities, and to support them to access HIV testing, STI services, HIV care and treatment and other health services. Nevertheless, the challenges of prevention and referral coverage persisted; coverage of HIV testing was just 65.6% among entertainment workers [28], 51% among men who have sex with men [29] and 53% among people who inject drugs [30].

#### Engaging health workers in difficult conditions to deliver quality services

Quality assurance of HIV services has been conducted through: (1) Continuum of Care coordination meetings; (2) clinical mentoring; (3) regional network meetings for clinicians and counsellors; (4) HIV drug resistance surveillance surveys, including monitoring for early warning indicators; (5) HIV/AIDS care and treatment symposia; and (6) roll-out of a continuous quality improvement (CQI) strategy. As part of the CQI strategy, operational districts are supported to collect indicators that measure the quality of patient management across the continuum of care, measure their own performance against these, and monitor and improve these measures as an integral part of their work. These activities were designed to maximize PLHIV retention in treatment. In 2011, retention in ART at 12, 24 and 60 months reached 92.6, 84.2 and 78.0% respectively [31].

One of the major constraints for government health services is the low civil servant remuneration. Health workers’ salary is well below subsistence, which tends to lead to under-the-table payments by patients and demotivated staff seeking to supplement their incomes with other work. NCHADS introduced a standard salary incentive scheme, the first of its kind in the health sector, which enhanced health worker motivation. The scheme was soon handed over to other operators as part of a broader performance-based financing and social health protection/insurance scheme linked to national health system strengthening efforts. More sustainable solutions are yet to be found [32].

#### Strengthening health service delivery systems

In addition to expanding HIV services and linking them with other relevant programmes, NCHADS made substantive efforts to strengthen health system. For example, since 2004, NCHADS has been working with 17 hospitals to integrate their multiple independent laboratories (i.e. those that had been established for HIV, STI, TB, malaria and other programmes). Merging them with the support of development partners into a single comprehensive “integrated laboratory” for the entire hospital created a model for subsequent nationwide expansion within the context of health system strengthening initiatives. Also, in 2005, when expansion of ART for children was planned, it was found that a majority of referral hospitals had no functioning paediatric outpatient or inpatient services. As this was largely due to lack of facilities and/or equipment and a shortage of paediatricians, NCHADS mobilized resources from development partners to build or renovate facilities and provide equipment to 22 hospitals in 2011. In the newly equipped and revitalized paediatric services, HIV care and treatment for children were introduced.

***Box 1.* How Cambodia Achieved Universal Access: Key Lessons Learned**

Cambodia was able to achieve universal access by:

1. Investing in strategic information for a clear understanding of the national HIV epidemic, to be able to deliver effective and efficient interventions;
2. Taking a vertical approach (e.g. 100% CUP) to controlling the epidemic in its early years;
3. Working with all partners to develop a single service delivery framework for preliminary learning and subsequent expansion of an evidence-based model rather than having many pilot projects and parallel programmes;
4. Involving the community meaningfully (as in PLHIV for Continuum of Care, and key populations for Continuum of Prevention to Care and Treatment), not only in service delivery but also policy formulation, programme design and monitoring and evaluation; and
5. Evolving from a vertical approach to the “linking” model as the epidemic and the response matured, and then adopting an integrated approach to maximize resources.

The early response of Phases I and II managed to curb the transmission that was driving Cambodia’s HIV epidemic (Box 1). As per Figure 1, HIV prevalence had declined from 1.7% (among adults aged 15–49) in 1998 to 0.7% in 2011, and the estimated number of new HIV infections plummeted from 20,000 annually in the early 1990s to around 1300 in 2012 [6]. New infections in children aged 0–14 years dropped from 1000 in 2000 to 100 in 2012.

#### Phase III. Towards the elimination of new HIV infections (2012–2020)

At the United Nations General Assembly High Level Meeting on AIDS in New York in June 2011, Cambodia expressed its support of the global goals and targets to intensify efforts to achieve the “Three Zeros.” As such, it has set an ambitious goal of eliminating new HIV infections by 2020 under the “Cambodia 3.0” framework [33]. The main components of this strategy are: (1) HIV prevention targeting key populations at higher risk, and linkages to health services; (2) Elimination of mother-to-child transmission (e-MTCT); and (3) Optimization of the cascade of interventions, with HIV testing, linkages to care and treatment, and ART as prevention (Figure 6).

**Figure 6:** Cambodia 3.0. A plan for the elimination of virtually all new HIV infections by 2020.

In continuing to strengthen prevention among key populations and improve their access to health services (component 1), a rapid response mechanism will be introduced in line with communicable disease outbreak investigation and control principles. The three functions of the mechanism are: (1) enhanced vigilance of potential transmission, risk behaviours and underperforming programmes; (2) targeted investigation of outbreaks and new pockets of risk, and assessment of the adequacy of the response; and (3) immediate strengthening of interventions (local micro-planning). Priority target populations at higher risk include people who inject drugs, people who use and/or inject drugs and sell sex, male and TG entertainment workers who sell sex, men who have sex with men frequenting saunas and bars, and partners of all these persons. Female entertainment workers who have more than seven clients a week are also an important population due to the large number of these women.

To accelerate timely HIV diagnosis among key populations (part of component 3), outreach HIV testing using finger-prick testing is being expanded by mobilizing NGOs and outreach workers. Finger-prick testing is also being introduced at most health centres where ANC and TB services are offered. The finger-prick testing is intended to streamline and simplify procedures for HIV diagnosis by significantly reducing referral steps, abolishing blood sample referral from health centres, and averting in-person referral from VCCT without pre-ART/ART. Drop-outs and delays are being mitigated through direct referral of all first test reactive cases found in the community to VCCT services co-located at pre-ART/ART sites. The same applies for first test reactive cases found at health facilities (including ANC and TB services at health centres).

Furthermore, integrated active case management is being introduced to maximize retention along the HIV cascades. A case management coordinator will be appointed in each operational district to ensure active follow-up of individuals from first test reactive in HTC through pre-ART/ART, PMTCT, TB/HIV and partner tracing and testing processes. He or she will be provided with case information regularly and authorized to mobilize and coordinate concerned service providers, HBC teams and outreach workers.

The current ART eligibility criteria are in line with the 2010 WHO guidelines [34], which recommend (1) starting ART at a CD4 count <350 cells/µl, (2) starting TB therapy immediately after diagnosis of TB with co-trimoxazole prophylaxis, and starting ART after two weeks of TB therapy regardless of CD4 count, (3) lifelong ART for pregnant women regardless of CD4 count (Option B+) which was introduced in late 2013, and (4) ART for prevention for the HIV-positive partner in serodiscordant couples at a CD4 count of 350–500 cells/µl, which was introduced in early 2014. As of 2014, NCHADS plans to adapt and adopt the 2013 WHO consolidated guidelines [35] for use of ARVs for treatment and prevention. Immediate treatment irrespective of CD4 count will also be considered for priority key populations at higher risk in line with the aforementioned rapid response mechanism.

Building on the past achievements in linking HIV, MCH and TB services and further strengthening health service delivery systems, the development of comprehensive linked/integrated service delivery models will be explored in collaboration with stakeholders engaged in sexual and reproductive health including adolescent health, non-communicable diseases, blood safety and hepatitis.

There are plans to strengthen surveillance, case reporting and programme monitoring systems. A unique identifier system is being piloted to enable the confidential tracking of service delivery to PLHIV, to strengthen case management and to enhance programme monitoring. Also, mathematical modelling and ad hoc studies will be conducted in order to monitor and assess the impact of innovative interventions, and to inform the adjustment of Cambodia 3.0 strategies.

### Remaining and emerging challenges

Challenges remain on both demand and supply sides of service delivery. Access to services by key populations remains constrained by a sub-optimal policy, legal and social environment. Cambodian health and community systems are still in a fragile state, with health workers chronically overloaded and receiving wages that have not kept pace with the cost of living. Despite strong leadership at national level, and although decentralization of HIV services during Phase II was successful, there is limited leadership and management capacity at sub-national level. Logistic and supply management of HIV-related medicines and other commodities is emerging as a significant concern. Without the strengthening of these systems, it will be difficult indeed to be able to verify whether and when there are zero new infections. It is equally crucial that the systems of data generation, analysis and use are robust, not only for surveillance but also for programming and budgeting purposes, and to inform policy dialogue and planning.

Compounding these challenges and potentially undermining the Cambodia 3.0 goals and aspirations is Cambodia’s marked dependence on international funding. In 2012, 87% of financial resources for the national response to HIV came from donors – mainly the Global Fund and the U.S. President’s Emergency Plan for AIDS Relief initiative [36]. Only 3% of general government expenditure on health was for HIV, while 38% of total external expenditure for health was expenditure for HIV/AIDS. International funds have been decreasing for the past three years, and given the unpredictability of external funding beyond 2015, it is now clear that domestic contributions [37, 38] must be steadily increased up to and beyond 2020. Accordingly, there is an urgent need to prepare a resource mobilization plan for sustained funding of essential HIV services to ensure that the elimination goal will be achieved and maintained [39].

## Conclusions

In May 2013, a National Health Sector HIV Program Review concluded that Cambodia was on track to achieving its goal of eliminating new HIV infections in the country by 2020. Indeed, combined, comprehensive prevention, care and treatment efforts and the linking of services have had a positive impact on the epidemic. Tens of thousands of new infections have been prevented, and tens of thousands of PLHIV have been given the prospect of a longer and more active life. Nevertheless, Cambodia must now protect the gains of the past amid on-going and emerging health system challenges and declining international funding. The approaches and linkages that have been developed over the past 20 years must be consolidated and at the same time remain flexible to respond effectively to the ever-changing nature of the epidemic. HIV services need to be integrated and mainstreamed gradually into the broader health system as the overall health system improves and new financing strategies are identified and implemented.

## Footnotes

1 Following political changes, namely the promulgation of the 2008 Law on the Suppression of Human Trafficking (see also part 4 of Phase II), there were significant changes in the number of women working in non-brothel-based entertainment establishments, and in the nature of transactional sex. “Entertainment workers” is the current term that defines women working in entertainment establishments who may or may not be selling sex.

## References

[1] The World Bank Group (2013). World development indicators [Internet]. http://data.worldbank.org/country/cambodia.

[2] WHO (2012). Cambodia country profile 2012.

[3] World Bank (2013). GDP per capita (current US$) [Internet]. http://data.worldbank.org/indicator/NY.GDP.PCAP.CD.

[4] National Institute of Statistics, Directorate General for Health, and ICF Macro (2011). Cambodia Demographic and Health Survey 2010.

[5] POLICY Project (2003). HIV/AIDS in the Mekong Region: Cambodia, Lao PDR, Thailand and Viet Nam: Current situation, future projections, socioeconomic impacts, and recommendations.

[6] National Center for HIV/AIDS, Dermatology and STD (2011). Estimations and projections of HIV/AIDS in Cambodia 2010–2015.

[7] National Center for HIV/AIDS, Dermatology and STD (2007). Report of HIV Prevalence among Drug Users.

[8] UNAIDS (2010). Cambodia takes MDG prize for excellence in its AIDS response [Internet]. http://www.unaids.org/en/resources/presscentre/featurestories/2010/september/20100920fsmdgcamboda-award/.

[9] Chaya N (2006). Cambodia and HIV: winning round two in a preventive fight. Popul Action Int.

[10] National Center for HIV/AIDS, Dermatology and STD (2006). Standard operating procedure for the outreach/peer education and 100% condom use program to sex workers in Cambodia.

[11] World Health Organization (2003). Review of the 100% condom use program in Cambodia: a report of findings and recommendations.

[12] National Center for HIV/AIDS, Dermatology and STD (2003). Continuum of care for people living with HIV/AIDS operational framework in Cambodia.

[13] Eang MT, Chheng P, Natpratan C, Kimerling M (2007). Lessons from TB/HIV integration in Cambodia. WHO Bull.

[14] National Center for HIV/AIDS, Dermatology and STD (2007). Standard operating procedures to initiate a linked response for prevention, care, and treatment of HIV/AIDS and sexual and reproductive health issues.

[15] National Center for HIV/AIDS, Dermatology and STD, National Center for Tuberculosis and Leprosy Control (2002). Framework for TB/HIV.

[16] Battambang Provincial Health Department, Family Health International/Cambodia, Gorgas TB Initiative at UAB (2008). TB screening and IPT initiation among HIV-infected persons, experience from Battambang Province. http://www.nac3.hacccambodia.org/documents/nac/Battambang_IPT_NAC_10Sep08-LF.pdf.

[17] National Center for HIV/AIDS, Dermatology and STD, National Center for Tuberculosis and Leprosy Control (2006). Standard operating procedures (SOP) for prompt testing of TB-HIV and rapid access to treatment and care services.

[18] Kingdom of Cambodia (2007). The law on suppression of human trafficking and sexual exploitation (Unofficial Translation), NS/RKM/0208/005.

[19] Ministry of Education, Youth and Sport (2010). Most-at-risk young people survey.

[20] Maher L, Mooney-Somers J, Phlong P (2013). Condom negotiation across different relationship types by young women engaged in sex work in Phnom Penh, Cambodia. Glob Public Health.

[21] Nishigaya K (2002). Female garment factory workers in Cambodia: migration, sex workers and HIV/AIDS. Women Health.

[22] National Center for HIV/AIDS, Dermatology and STD (2008). Second quarterly comprehensive report, 2008: HIV/AIDS & STI prevention and care programme.

[23] Page K, Stein E, Sansothy N (2009). Sex work and HIV in Cambodia: trajectories of risk and disease in two cohorts of high-risk young women in Phnom Penh, Cambodia. BMJ Open.

[24] Pearson E, Robertson P, Colm S (2010). Off the streets: arbitrary detention and other abuses against sex workers in Cambodia.

[25] United Nations Inter-Agency Project on Human Trafficking Cambodia: Exodus to the Sex Trade? Effects of the global financial crisis on women’s conditions and opportunities [Internet]. http://www.no-trafficking.org/reports_docs/siren/siren_cb-04.pdf.

[26] National AIDS Authority (2012). Cambodia Country Progress Report 2012: monitoring the progress towards the implementation of the declaration of commitment on HIV and AIDS.

[27] National Center for HIV/AIDS, Dermatology and STD (2010). Standard operating procedures for a continuum of prevention to care and treatment for female entertainment workers, men who have sex with men and transgenders in Cambodia.

[28] National Center for HIV/AIDS, Dermatology and STD (2013). 2011 Cambodia Sexually Transmitted Infections Survey: Female Entertainment Workers.

[29] FHI 360 (2010). Behavioral Risks On-Site Serosurvey among At-Risk Urban Men in Cambodia.

[30] National Center for HIV/AIDS, Dermatology and STD, and National Authority for Combating Drugs (2008). Integrated behavioral and biological sentinel survey: HIV prevalence among drug users 2007.

[31] National Center for HIV/AIDS, Dermatology and STD (2012). Annual Report 2011.

[32] Lowe D, Himelfarb T, Roberts J, Pick B, Berk S (2011). HIV/AIDS Portfolio Evaluation, USAID/Cambodia.

[33] National Center for HIV/AIDS, Dermatology and STD (2012). Conceptual framework for elimination of new HIV infections by 2020.

[34] World Health Organization (2010). Antiretroviral therapy for HIV infection in adults and adolescents.

[35] World Health Organization (2013). Consolidated guidelines on the use of antiretroviral drugs for treating and preventing HIV infection.

[36] National AIDS Authority (2011). National AIDS spending assessment 2009–2010.

[37] Bureau of Health Economics and Financing, Department of Planning and Health Information (2008). Strategic framework for health financing 2008–2015.

[38] Medical Xpress (2010). New report finds Cambodia’s HIV/AIDS fight at critical crossroads in funding, prevention. Phys.org [Internet].

[39] Macan-Markar M (2010). Cambodia: aid dependence May Hurt successes in HIV, AIDS.
